# Values of D-dimer, APTT, PT and INR in distinguishing between acute ischemic stroke patients with and without non-valvular atrial fibrillation

**DOI:** 10.5937/jomb0-60388

**Published:** 2026-03-17

**Authors:** Qing Du, Xiaohui Zhao, Haixia Song, Ju Jiang, Kaiming Zheng, Yuezhan Zhang

**Affiliations:** 1 Lianyungang Affiliated Hospital of Nanjing University of Chinese Medicine, Department of Neurology, Lianyungang, China; 2 Lianyungang Affiliated Hospital of Nanjing University of Chinese Medicine, Department of Laboratory, Lianyungang, China; 3 Lianyungang Affiliated Hospital of Nanjing University of Chinese Medicine, Department of Geriatrics, Lianyungang, China; 4 Jiangsu Province Hospital of Chinese Medicine, Department of Geriatrics, Nanjing, Jiangsu, China

**Keywords:** coagulation function, diagnosis, acute ischemic stroke, non-valvular atrial fibrillation, koagulaciona funkcija, dijagnoza, akutni ishemijski moždani udar, nevalvularna atrijalna fibrilacija

## Abstract

**Background:**

Treatment strategies differ in acute ischemic stroke (AIS) patients with and without non-valvular atrial fibrillation (NVAF): AIS with NVAF requires anticoagulation (e.g. warfarin/direct oral anticoagulants) for secondary prevention, while AIS without NVAF relies on antiplatelet therapy (e.g. aspirin/clopidogrel). It remains unclear whether coagulation function indices can be used to distinguish between the two conditions.

**Methods:**

In this retrospective study, AIS patients with NVAF (AIS with NVAF group, n=43) and without NVAF (AIS without NVAF group, n = 90) admitted to our hospital were enrolled for final analysis. The levels of coagulation function indices, including d-dimer (DD), activated partial thromboplastin time (APTT), prothrombin time (PT), international normalised ratio (INR), thrombin time (TT), and fibrinogen, were tested before or on admission and compared between the two groups.

**Results:**

Compared with AIS without NVAF group, DD, PT, and INR were increased and APTT was decreased in AIS with NVAF group (P&lt; 0.05), whereas, there were no significantly statistical differences of TT and fibrinogen (P&gt; 0.05); next, the AUC in distinguishing between AIS with and without NVAF were 0.753, 0.761, 0.775, and 0.770 for DD, APTT, PT, and INR, respectively (P&lt; 0.05).

**Conclusions:**

DD, APTT, PT and INR demonstrated moderate diagnostic value (AUC 0.75-0.78) for distinguishing AIS with NVAF.

## Abstract (sr)

**Uvod:**

Strategije lečenja razlikuju se kod pacijenata sa akutnim ishemijskim moždanim udarom (AIMU) u zavisnosti odprisustva nevalvularne atrijalne fibrilacije (NVAF). KodAIMU sa NVAF potrebna je antikoagulantna terapija (npr.varfarin ili direktni oralni antikoagulansi) za sekundarnuprevenciju, dok se kod AIMU bez NVAF primenjuje antiagregaciona terapija (npr. aspirin ili klopidogrel). Jos uveknije jasno da li se pokazatelji koagulacione funkcije mogukoristiti za razlikovanje ova dva stanja.

**Metode:**

U retrospektivnu studju su ukjučeni pacjentiprimjeni u nasu bolnicu sa AIMU i NVAF (grupa AIMU saNVAF, n=43) i bez NVAF (grupa AIMU bez NVAF, n = 90).Ispitivani su nivoi pokazatelja koagulacione funkcije: Ddimer (DD), aktivirano parcij'alno tromboplastinsko vreme(APTT), protrombinsko vreme (PT), internacionalni normalizovani odnos (INR), trombinsko vreme (TT) i fibrinogen. Vrednosti su merene pre prijema ili pri prijemu, azatim upoređene između grupa.

## Introduction

Acute ischemic stroke (AIS) is caused by occlusion of cerebral arteries, accounting for about 87% of all strokes [1][2]. Non-valvular atrial fibrillation (NVAF) is one of the common complications of AIS [3]. Secondary prevention strategies with anticoagulant drugs for AIS with NVAF and antiplatelet medications for AIS without NVAF have still been widely used. In conclusion, treatment strategies differ in AIS patients with and without NVAF. How to distinguish AIS patients with and without NVAF remains one of the most challenging and intractable problems for clinical physicians. At this time, whether there are easy-to-obtain and inexpensive blood tests that can help differentiate between the two conditions has become a topic of great interest for clinicians when they encounter patients with AIS.

The pathophysiological basis of the thromboembolic complications in NVAF entails the presence of a hypercoagulable state [4]. At present, D-dimer (DD) is still one of the important coagulation indicators reflecting coagulation function, fibrinolytic function or both activation [5]. Studies have confirmed that DD levels are significantly increased in patients with atrial fibrillation (AF) [6][7]. In addition, DD was shown to be increased dramatically in the NVAF with stroke group compared to the NVAF group [8].

To the best of our knowledge, whether coagulation function indices can be used to distinguish between AIS patients with and without NVAF remains unclear. Although there have been some related studies, they mainly focus on a single marker [9] and lack a systematic analysis of multiple coagulation function indicators. Our study will confirm for the first time whether the parameters of coagulation function can accurately distinguish AIS patients with and without NVAF.

## Materials and methods

### Study population

**Figure 1:**
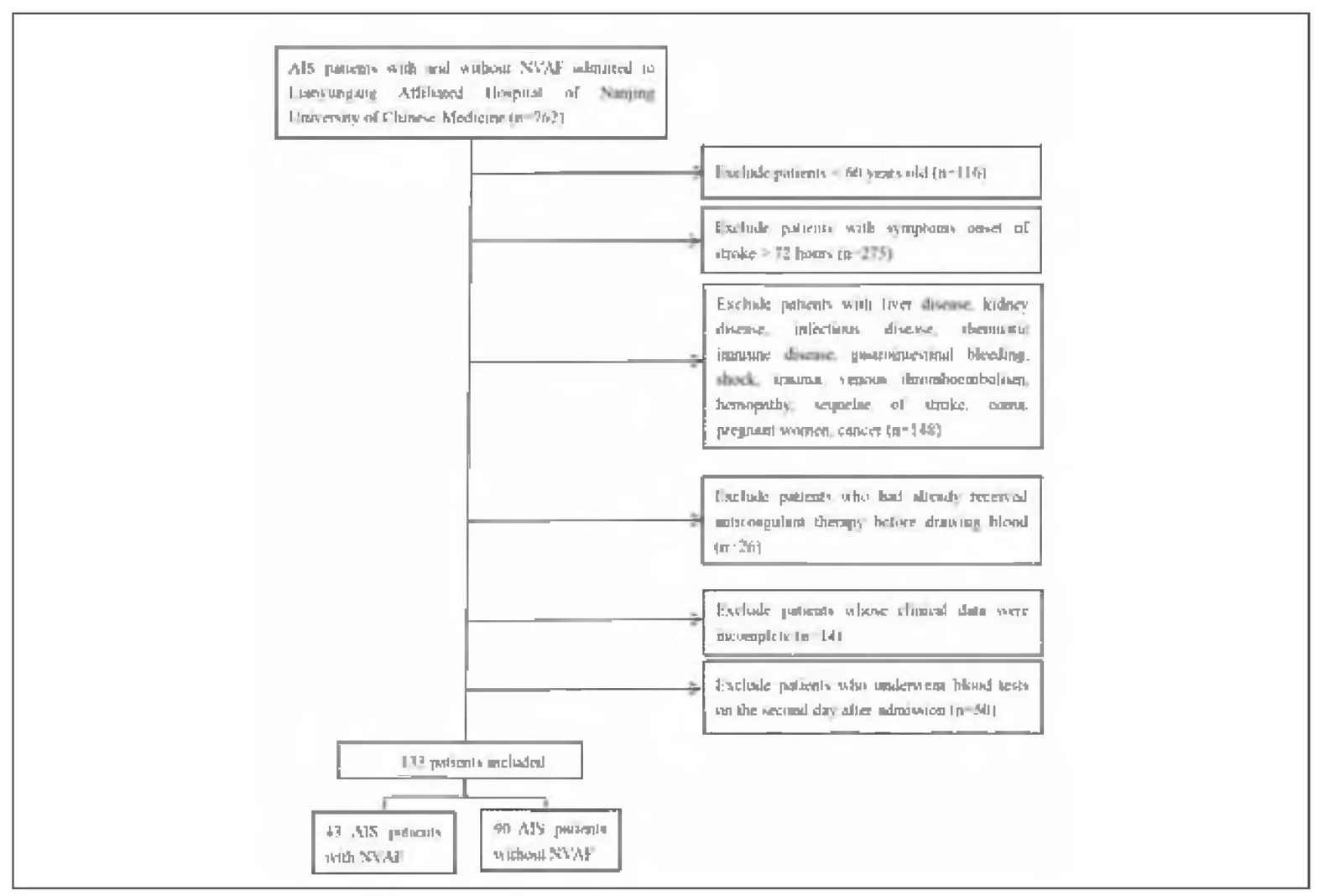
The screening process of the study subjects. Of the 762 patients, 133 were identified. AIS - acute ischemic stroke, NVAF - non-valvular atrial fibrillation

A total of 133 AIS patients from Lianyungang Affiliated Hospital of Nanjing University of Chinese Medicine from January 2022 to December 2024 were enrolled in this study. The diagnoses of AIS and AF were performed following the relevant guidelines [10], respectively. All patients underwent coagulation function, electrocardiogram, computed tomography or magnetic resonance imaging, if necessary. The flow chart of study patients is shown in Figure 1. Sample size was calculated using Power Analysis (PASS 15.0) with α=0.05, β=0.2, based on the DD mean difference (0.26 mg/L) from pilot data. The minimum required sample was 38 per group. In addition, a further 10% possibility of shedding needs to be considered.

Inclusion criteria were adults ≥60 years old; symptoms of stroke onset within 72 hours. Exclusion criteria were patients with liver disease, kidney disease, infectious disease, rheumatic immune disease, gastrointestinal bleeding, shock, trauma, venous thromboembolism, hemopathy, sequelae of stroke, coma, pregnant women, cancer, and patients who had already received anticoagulant therapy before drawing blood.

The study was conducted following the Declaration of Helsinki and approved by the Ethics Committee of Lianyungang Affiliated Hospital of Nanjing University of Chinese Medicine (No.2025-KY-063).

### Coagulation function test

Platelet-depleted plasma was obtained by standard centrifugation at 1500xg for 15 minutes. An immunoturbidimetric assay quantified DD. Coagulation function indices were detected using a CS-5100 coagulation analyser (Sysmex, Germany), including activated partial thromboplastin time (APTT), prothrombin time (PT), international normalised ratio (INR), thrombin time (TT), and fibrinogen. A total of 4 mL blood samples were collected from the patient's fasting elbow vein (using a sodium citrate anticoagulant tube), and the results were obtained automatically after the machine started (blood samples were collected before any antithrombotic therapy within 1 hour of admission).

Quality control: at least 2 levels of quality control (normal, abnormal) were run before testing every day, covering the clinical reference range. Quality control results were recorded and analysed using Levey-Jennings plots or Westgard multiple rules (e.g., 1-2s warnings, 1-3s out of control). If it is out of control, the cause (reagent failure, instrument failure, operation error, etc.) should be investigated, and the test should be recalibrated or re-measured after replacing the reagent. Clean the reaction cup and sampling needle every day, and check the liquid system (whether the reagent and water are unobstructed). Instrument performance verification (e.g., coagulation time repeatability, linear range) is performed regularly (e.g., monthly).

### Statistical analysis

Statistical analysis was performed using SPSS 19.0 software. Descriptive analyses for continuous variables were used to calculate mean values and standard deviations. The qualitative data were compared with a chi-square test. The student's *t*-test of independent samples was used to compare the means. Prognostic performance was tested by calculation of the receiver operating characteristic (ROC) curve and displayed in the Area under the curve (AUC). According to the maximum Youden index, the corresponding cut-off value, and the diagnostic sensitivity and specificity under this cut-off value were determined. P<0.05 was considered statistically significant.

## Results

### Characteristics of the total population

Comparison of the data of the two groups showed that there was no significant difference in age, gender and other data (P>0.05), except for the rates of diabetes mellitus and coronary heart disease between AIS patients with and without NVAF, respectively (P<0.05) (Table 1).

**Table 1:** Comparisons of the levels of different variables between the two groups.

	AIS with the NVAF group<br>(n=43)	AIS without the NVAF group<br>(n=90)	Statistics	p value
Age (years)	73.98 ± 6.55	72.22 ± 8.01	t=1.249	0.214
Gender [Male, n (%)]	21 (48.8%)	59 (65.6%)	2=3.393	0.065
Hypertension [n (%)]	27 (62.8%)	70 (77.8%)	2=3.311	0.069
Diabetes mellitus [n (%)]	9 (20.9%)	36 (40.0%)	2=4.727	0.030
Coronary heart disease [n (%)]	13 (30.2%)	10 (11.1%)	2=7.438	0.006
NIHSS score (point)	4.26 ± 2.91	3.42 ± 1.87	t=1.715	0.092

### Comparisons of coagulation function indices between the two groups

**Figure 2:**
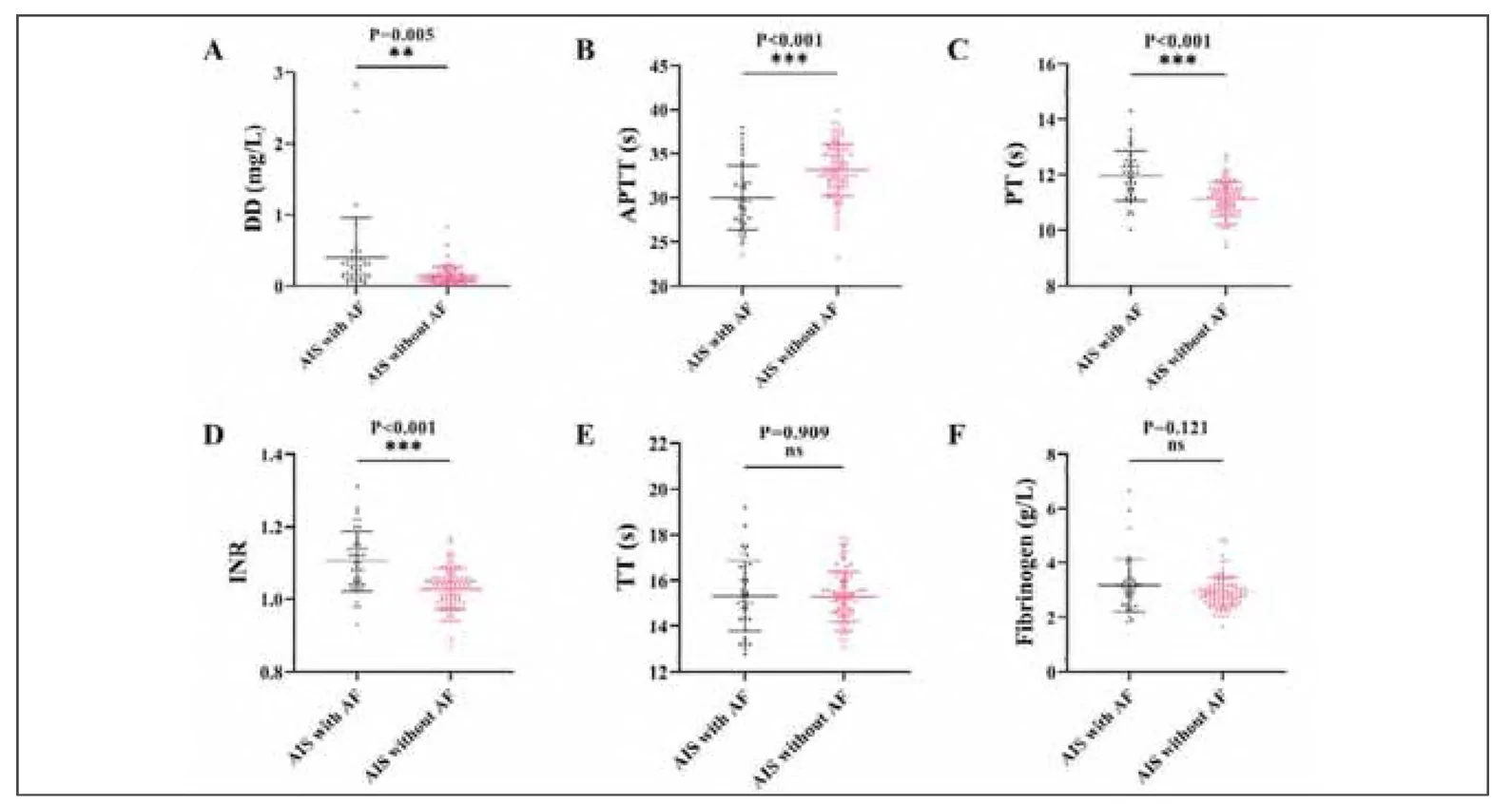
Comparisons of coagulation function indices between the two groups. A: Comparison of DD. B: Comparison of APTT C: Comparison of PT D: Comparison of INR. E: Comparison of TT F: Comparison of fibrinogen. ** indicates P<0.01, *** indicates P<0.001, ns represents P>0.05.AIS - acute ischemic stroke, NVAF - non-valvular atrial fibrillation, DD - d-dimer, APTT - activated partial thromboplastin time, PT - prothrombin time, INR - international normalised ratio, TT - thrombin time.

The levels of DD, PT, and INR were significantly increased, and APTT was significantly decreased in the AIS with NVAF group compared to the AIS without NVAF group (P<0.05). In contrast, there were no significant differences in TT and fibrinogen (P>0.05, Figure 2).

### Comparisons of ROC curves of DD, APTT, PT, and INR in distinguishing between AIS patients with and without NVAF

**Figure 3:**
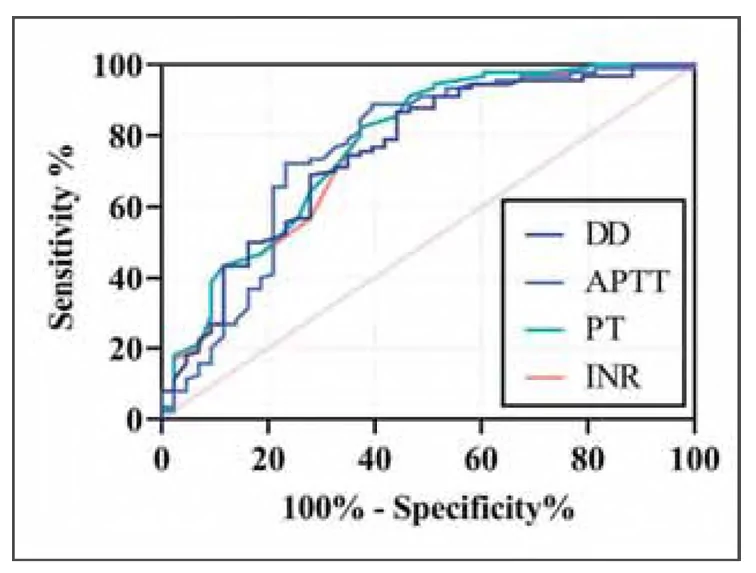
Comparisons of ROC curves of DD, APTT PT, and INR in distinguishing between AIS patients with and without NVAF. AIS - acute ischemic stroke, NVAF - non-valvular atrial fibrillation, DD - d-dimer, APTT - activated partial thromboplastin time, PT - prothrombin time, INR - international normalised ratio.

The results of ROC curve analysis showed that the efficacy of DD, APTT, PT and iNr in identifying the presence or absence of NVAF in AIS patients was shown in Figure 3 and Table 2. The AUC of the ROC curve was 0.753-0.775, which had a high clinical reference value.

**Table 2:** Diagnostic performance of coagulation indices.

Parameter	AUC (95%CI)	Cut-off	Sensitivity (%)	Specificity (%)	Youden Index
DD	0.753 (0.671 to 0.823)	0.21 mg/L	86.7	55.8	0.425
APTT	0.761 (0.680-0.831)	29.8 s	60.5	88.9	0.494
PT	0.775 (0.695-0.843)	11.6 s	62.8	82.2	0.450
INR	0.770 (0.689-0.839)	1.07	62.8	82.8	0.450

## Discussion

DD, APTT, PT, INR, TT and fibrinogen were selected in this study because they cover the whole process of coagulation and fibrinolysis. Among them, APTT/PT, as the cornerstone index of the endogenous/exogenous coagulation pathway, is sensitive to a hypercoagulable state. INR is a standardised PT value to avoid the influence of reagent differences. DD directly reflects fibrin degradation and is a specific marker of thrombosis. Although APTT/PT is not a specific index of NVAF, its changes suggest the overall activation state of the coagulation system. In AF patients, the combination of tissue factor release from atrial endothelial injury (exogenous pathway) and blood stasis (endogenous pathway) leads to thrombin burst production, which provides a pathophysiological basis for the differential value of APTT/PT.

In the present study, we found that DD was significantly increased in the AIS with NVAF group compared with the AIS without NVAF group. The previous study result by Liu et al. [11] was consistent with our conclusion, confirming a statistical difference in DD between AIS groups with and without NVAF. Other researchers also came to a similar conclusion as they observed that DD in patients with cardioembolic stroke were significantly higher as compared with non-cardioembolic stroke [12]. However, compared with the study by Liu et al. [11], the AUC of DD in the current study was only lower, which may be due to the exclusion of patients with mild AIS (NIHSS<5) in the study by Liu et al. [11]. Of course, this idea still needs to be verified by a large number of cases. The increase of DD reflects the specific thrombophilia of atrial fibrillation, which may be related to the following mechanisms: ① atrial blood stasis (Virchow triad) promotes fibrin deposition [13]; ② Platelet-leukocyte aggregates release procoagulant microparticles [14]; ③ Endothelial injury leads to high expression of tissue factor [15]. The AUC in distinguishing between AIS patients with and without NVAF was 0.753 for DD, which further confirmed the accuracy and reliability of discrimination performance.

APTT is mainly used to assess the function of the endogenous coagulation system [16]. Huang et al. [17] found that APTT was significantly shorter in patients with AIS compared with controls, indicating that AIS presented a hypercoagulable state. In the present study, we also discovered that APTT was decreased considerably in the AIS with NVAF group compared with the AIS without NVAF group, suggesting that APTT could also be used to distinguish between them. The AUC in distinguishing between AIS patients with and without NVAF was 0.761 for APTT, confirming that it was a valuable tool for screening for AIS with NVAF. There is a possible interpretation as to the difference in APTT between AIS with and without NVAF, which is associated with different hypercoagulable states caused by AIS of different etiologies [18].

In addition, we also found that PT was significantly increased in the AIS with NVAF group compared with the AIS without NVAF group, suggesting that PT could be used to differentiate between AIS with and without NVAF. The AUC in distinguishing between them was 0.775 for PT, which further verified the feasibility of discrimination performance. Mathematical conversion of INR by PT showed the same trend as INR has been proved to be used to distinguish between AIS with and without NVAF. The AUC in distinguishing between them was 0.770 for INR, which further confirmed that it was a valuable tool for detecting AIS with NVAF. As for TT and fibrinogen, we found that they could not be used to distinguish between AIS with and without NVAF. Elevated PT/INR reflects activation of the extrinsic pathway, likely due to: Increased tissue factor exposure from atrial endothelial damage [19]; or warfarin-like effect of inflammatory cytokines in NVAF [20]. However, these mechanisms need to be verified by further studies.

This study found that routine coagulation screening indicators (APTT/PT/INR) had important clinical significance in the differential diagnosis of NVAF, and revealed their role in the etiological classification of stroke. The shortened APTT reflects the activation of the endogenous coagulation pathway, suggesting the existence of a hypercoagulable state in NVAF patients. Prolonged PT/INR was associated with tissue factor exposure, consistent with the thrombosis mechanism of AF Although DD is a fibrinolytic marker, the combination of APTT/PT/INR as an inexpensive and rapid screening test can provide a bedside identification tool for primary hospitals, especially for patients with paroxysmal atrial fibrillation that is difficult to capture by electrocardiogram. Based on the results of this study, we believe that the risk of NVAF can be assessed by testing the coagulation function in AIS patients in the future. At the same time, according to the threshold of coagulation function indicators, corresponding measures should be taken promptly. For example, for AIS patients with DD>0.21 mg/L + PT>11.6s Urgent ECG screening. If APTT<29.8s Prioritise anticoagulation assessment.

However, the sample size from a single centre was small (only 133 subjects, and only 43 in the AIS with NVAF group). Second, it was a retrospective analysis and thus lacked a more rigorous design. In the future, we need to carry out more in-depth and comprehensive research and analysis according to the above limitations as soon as possible.

## Conclusion

DD, APTT, PT and INR may help to distinguish whether AIS is complicated with NVAF, and provide a reference for optimising antithrombotic regimens.

## Dodatak

### Acknowledgements

This study was supported by the Grants from Jiangsu Province 333 High Level Talent Training Project, Lianyungang Ageing Health Research Project (No. L202319), and Lianyungang Traditional Chinese Medicine Administration Project (No. LZYYB202403). The sponsors had no involvement in the study design, data interpretation, or publication decisions.

### Data availability

Data are available on request from the corresponding author.

### Conflict of interest statement

All the authors declare that they have no conflict of interest in this work.
